# What Is the Purpose of the Orphan Drug Act?

**DOI:** 10.1371/journal.pmed.1002191

**Published:** 2017-01-03

**Authors:** Matthew Herder

**Affiliations:** Health Law Institute, Faculties of Medicine and Law, Dalhousie University, Halifax, Nova Scotia, Canada

## Abstract

Matthew Herder suggests it may be time to re-examine the purpose of the U.S. Orphan Drug Act.

The Orphan Drug Act (ODA) [1], first enacted in the United States in 1983, was set up to encourage the development of drugs for rare diseases. At that time, drug therapies for such diseases were rarely developed. Three decades later, a growing proportion of industry research and development (R&D) [2] and regulatory drug approvals [3] target diseases affecting fewer than 200,000 persons in the United States, the prevalence-based threshold of rare disease under the ODA.

In a new article published in *PLOS Medicine*, Aaron Kesselheim and colleagues document an embedded trend: within the increasing number of drug approvals targeting rare diseases, there is a substantial minority of biomarker-defined subsets of more common diseases, especially cancers [4]. Commentators have long worried about this phenomenon of “salami slicing” common diseases for the purposes of drug approval because of the market advantages that orphan drug status confers [5,6]. Due to lower R&D costs (e.g., relatively small clinical trials or observational studies), expedited regulatory reviews, and minimal competition even after patent and ODA market protection expire, rare-disease-targeting orphan drugs are now amongst the most expensive and profitable drugs on the market in the world [7].

Most of the policy and scholarly response to the orphan drug pricing problem to date has been to explore new ways to evaluate orphan drug performance following regulatory approval [8,9]. However, there is little indication that health care payers are successfully pushing back on drug price points. Meanwhile, the findings of Kesselheim and colleagues underscore how the operation of the ODA upstream—at the point of regulation—serves to expand the scope of the problem. With the increasing ability to more precisely identify biomarker-defined subsets of disease, it is perhaps time to re-examine how the ODA distinguishes rare versus common forms of disease or, even more fundamentally, what the ODA is meant to achieve.

## The Definition of an Orphan

Before the ODA became law, Congress heard diverse views about which R&D “orphans” the legislation should attempt to rescue [10]. Some witnesses focused on rare diseases during Congressional hearings, whereas others advocated for “orphan medical devices and medically necessary foods” [10]. Still others spoke in favor of “drugs for less developed countries,” or vaccines, which manufacturers had moved away from due to high product liability concerns at that time. Worried that this lack of consensus might undermine the bill’s progress, the ODA’s authors made a political choice to focus on rare diseases [10].

Even so, the ODA did not originally include a prevalence-based definition of rare disease. Rather, the ODA defined a “rare disease or condition” as one that “occurs *so infrequently* in the United States that there is no reasonable expectation that the cost of developing and making available in the United States a drug for such disease or condition will be recovered from the sales in the United States of such drug” [11]. Orphan drug status was therefore not granted simply because it targeted a rare disease; rather, the disease had to be rare enough to occasion market neglect.

However, just as the Food and Drug Administration (FDA) began making “determinations” about whether a given disease met the ODA’s test of market neglect, the FDA’s task was grossly simplified. In 1984 the ODA was amended, redefining rare diseases as those affecting “less than 200,000 persons in the United States” (the prevalence-based definition) or more than 200,000 persons, but for whom “there is no reasonable expectation that the cost of developing and making available in the United States a drug for such disease or condition will be recovered from the sale in the United States” (a commercial viability definition) [12].

With that change, the FDA went from requiring evidence of the commercial non-viability of orphan drug R&D to assuming commercial non-viability, provided the drug targeted fewer than 200,000 persons. Numerous studies that suggest that orphan drugs are actually more profitable than non-orphan drugs call this underlying assumption into serious question [7,13,14]. Avoiding any accounting of their actual R&D costs, nearly all of the more than 2,000 orphan drug designations sought and obtained by drug-makers between 1983 and 2011 fall within the prevalence-based definition [15]. And without upfront scrutiny of the relationship between disease prevalence and anticipated profits, drug manufacturers are routinely able to price orphan drugs at US$100,000–US$200,000 per patient per year, needing only 5,000–10,000 patients to generate US$1 billion in annual revenues.

## Revise and Reclaim the ODA

What should be done? One idea is for the FDA to try yet again to cut down on the practice of salami slicing and, in turn, to better discriminate between genuine and artificial rare diseases. In 1992, the FDA first purported to curb salami slicing by requiring that, for subsets of common diseases to be considered rare, they needed to be “medically plausible” [16], a term it failed to define. Twenty-one years later, the FDA finally promulgated more promising regulations [17] that hold drug manufacturers to a higher standard of evidence. When seeking an orphan drug indication, manufacturers must now show not only why one subset of a disease should be targeted by their drug, but also why the drug is inappropriate outside the selected subset [18]. However, the findings of Kesselheim and colleagues [4] suggest that these new regulations—or the FDA’s application of them—may not be adequate to the task. Another potentially more fruitful approach is to limit orphan drug designation to disease pathways rather than the rarity of the disease per se [4].

Fundamentally, though, the purpose of the ODA merits re-examination. At bottom, the ODA was intended to redistribute resources to medical needs that would otherwise be marginalized by market forces. With the introduction of the prevalence-based definition of rare disease, we began losing sight of the ODA’s core, redistributive function.

To restore that function, we need to open up the very concept of an orphan disease or condition under the ODA and resume the scrutiny of claims of market-mediated, unmet medical need instead of policing the ever-shifting boundaries of disease. After all, “the category of orphan diseases bears no essential relationship to their prevalence, morbidity, or mortality” [19]. Markets also discourage a range of other research areas, including research involving pregnant women (because of perceived risks to the foetus) [20], comparative effectiveness research that seeks to assess the risks and benefits of competing drug treatments (because of the difficulty involved in patenting that type of information) [21], and research into diseases that disproportionately affect the world’s poor (because of the population’s low purchasing power) [22]. All of these areas of research carry tremendous social welfare gains; however, they have been effectively orphaned by the ODA’s current focus on rare diseases. Policymakers can reclaim the ODA by removing the prevalence-based definition of a rare disease, allowing other areas of research to qualify as orphans and reviving the FDA’s original, albeit short-lived, task of scrutinizing the demonstrable level of market neglect of the affected population, whether for reasons of rarity, poverty, gender, or otherwise. Waiting to see what comes down the pipe and then attempting to negotiate better prices for orphan drugs seems unlikely to succeed as a strategy for securing access to marginalized, but socially valuable, health innovations.

## Abbreviations

FDA: Food and Drug Administration
ODA: Orphan Drug Act
R&D: research and development
